# ARF6 promotes hepatocellular carcinoma proliferation through activating STAT3 signaling

**DOI:** 10.1186/s12935-023-03053-y

**Published:** 2023-09-16

**Authors:** Yabing Hu, Yongchu Huang, Xiaohang Xie, Longshan Li, Yong Zhang, Xiaochao Zhang

**Affiliations:** 1 Department of Dermatology, Tongji Hospital, Tongji Medical College, Huazhong University of Science and Technology, Wuhan, China; 2 Department of Laboratory Medicine, Wuhan No.1 Hospital, Wuhan, China

**Keywords:** Hepatocellular carcinoma, ARF6, STAT3, Proliferation, Therapy

## Abstract

**Background:**

Hepatocellular Carcinoma (HCC) possesses the high mortality in cancers worldwide. Nevertheless, the concrete mechanism underlying HCC proliferation remains obscure. In this study, we show that high expression of ARF6 is associated with a poor clinical prognosis, which could boost the proliferation of HCC.

**Methods:**

Immunohistochemistry and western blotting were used to detect the expression level of ARF6 in HCC tissues. We analyzed the clinical significance of ARF6 in primary HCC patients. We estimated the effect of ARF6 on tumor proliferation with in vitro CCK8, colony formation assay, and in vivo nude mouse xenograft models. Immunofluorescence was conducted to investigate the ARF6 localization. western blotting was used to detect the cell cycle-related proteins with. Additionally, we examined the correlation between ARF6 and STAT3 signaling in HCC with western blotting, immunohistochemistry and xenograft assay.

**Results:**

ARF6 was upregulated in HCC tissues compared to adjacent normal liver tissues. The increased expression of ARF6 correlated with poor tumor differentiation, incomplete tumor encapsulation, advanced tumor TNM stage and poor prognosis. ARF6 obviously promoted HCC cell proliferation, colony formation, and cell cycle progression. In vivo nude mouse xenograft models showed that ARF6 enhanced tumor growth. Furthermore, ARF6 activated the STAT3 signaling and ARF6 expression was positively correlated with phosphorylated STAT3 level in HCC tissues. Furthermore, after intervening of STAT3, the effect of ARF6 on tumor-promoting was weakened, which demonstrated ARF6 functioned through STAT3 signaling in HCC.

**Conclusions:**

Our results indicate that ARF6 promotes HCC proliferation through activating STAT3 signaling, suggesting that ARF6 may serve as potential prognostic and therapeutic targets for HCC patients.

**Supplementary Information:**

The online version contains supplementary material available at 10.1186/s12935-023-03053-y.

## Introduction

As one of the most universal malignant tumors, hepatocellular carcinoma (HCC) ranks the fourth, in the leading causes of cancer related mortality worldwide [1]. Although it is generally believed that a curable treatment for HCC is still surgical operation, HCC patients have a poor prognosis as before [1, 2] [3]. For HCC patients, effective methods in early diagnosis are lacking, which leads to high mortality and poor prognosis. Because of the fuzzy molecular mechanism of the progression of HCC, effective treatment targets are deficient for HCC patients. Therefore, it is necessary to investigate the potential molecular mechanism of HCC progression, which contributes to developing better treatments.

As a member of the ARF family, ADP ribosylation factor 6 (ARF6) belongs to the Ras superfamily of small GTP-binding proteins. ARF6 is reported to localize in the cytomembrane and endosomes [4, 5]. It is well demonstrated that ARF6 recycles between GTP-bound (active) and GDP-bound (inactive) forms. Guanine nucleotide exchange factors (GEFs) and GTPase-activating proteins (GAPs) are involved in controlling the balance between the two states [4]. Researchers have identified 15 GEFs in humans and classified them into 6 families (Cytohesin 1–4, EFA6A-D, BRAG1-3, BIG1/2, GBF, FBX8). A common Sect. 7 domain exists in all GEFs. It is revealed that 28 GAPs are confirmed and sorted into 10 families (ArfGAP1, ARFGAP2/3, ADAP1/2, SMAP1/2, AGFG1/2, GIT1/2, ASAP1-3, ACAP1-3, ARAP1-3, AGAP1-11) [6]. ARF6 is involved in biological processes and plays key roles, such as actin cytoskeletal rearrangements [4, 5]. ARF6 regulates endocytosis and the recycling of some membrane receptors, for example, EGFR [7]. Moreover, it is previously reported that ARF6 has important effects on proliferation, angiogenesis, invasion, and metastasis, microvesicle formation in various cancers [8–14]. As a molecular switch, ARF6 could activate downstream signaling pathways in cells. However, the role of ARF6 in HCC proliferation remain obscure.

In cells, janus kinase 2 (JAK2)-signal transducer and activator of transcription 3 (STAT3) signaling pathway belongs to one of the important signaling pathways, and it is involved in cell proliferation and differentiation by downstream effector factors regulation [15]. JAK2/STAT3 signaling pathway activation takes key parts in carcinogenesis and progression of different kinds of cancers. It helps to form tumor inflammatory microenvironment, resulting in the tumorigenesis and progression of many cancers in human. JAK2/STAT3 signaling pathway is widely demonstrated to highly abnormally activate in various cancers, for example, pancreatic cancer [16, 17], gastric cancer [18, 19], breast cancer [20–22], liver cancer [23–25], colorectal cancer [26, 27], colon cancer [28, 29], ovarian cancer [30, 31], lung cancer [32–34]. In glioma cells, the phosphorylation of STAT3 and JAK2 is obviously increased, which contributes to the proliferation-promoting and the apoptosis-inhibiting in glioma cells [35, 36]. The activation of JAK2/STAT3 signaling pathway causes the reduction of the adriamycin-induced aging of cells, and obviously promotes liver cancer cells proliferation [37]. Mesenchymal stem cells could conduce to tumor formation by IL-6/JAK2/STAT3 pathway in lung cancer [34]. JAK2/STAT3 pathway could act as key targets for screening anti-tumor drugs [38]. Furthermore, targeting this pathway could obviously inhibit cancer progression [39–41].

In this study, we conclude that ARF6 could enhance the proliferation and growth of HCC cells through activating STAT3 signaling pathway, which suggests that ARF6 may serve as potential prognostic and therapeutic targets for HCC patients.

## Results

### ARF6 is highly expressed in HCC, and high ARF6 expression correlates with poor prognosis

**Fig. 1:**
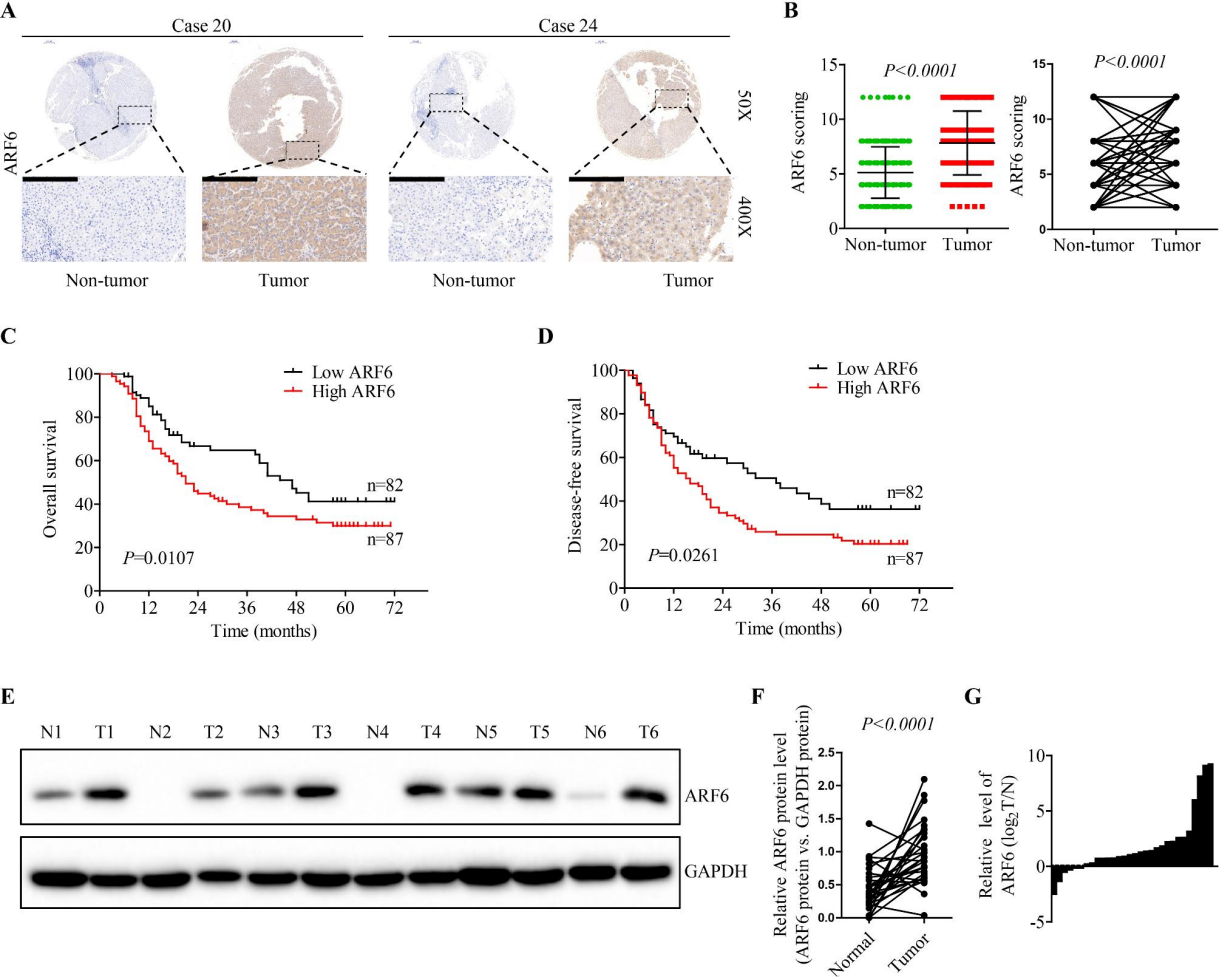
**ARF6 is highly expressed in HCC, and elevated ARF6 expression correlates with poor prognosis. (A, B)** Immunohistochemical staining (IHC) and expression scoring of ARF6 was conducted in 169 HCC tissues. Representative pictures were shown (scale bar: 200 μm). **(C, D)** Kaplan-Meier analysis was applied to demonstrate the correlation between ARF6 expression and overall survival or disease-free survival of HCC patients. The cutoff for distinguishing low or high ARF6 expression was the median value. **(E)** The protein level of ARF6 was analyzed in additional 136 paired HCC tissues (tumor, T) with corresponding adjacent non-cancerous tissues (normal, N) by western blot. Representative western blot results were shown. **(F)** Statistical analysis showed that ARF6 was upregulated in HCC tissues, compared with that in normal liver, where GAPDH was used as a control. **(G)** Statistical analysis showed that ARF6 bands of HCC tissues were quantified and shown in the bar chart after being normalized to the respective adjacent non-tumor tissues

To study the clinical significance of ARF6 in hepatocellular carcinoma, we analyzed the level of ARF6 in HCC patients with Online Oncomine dataset. We found that ARF6 mRNA was obviously higher in HCC tissues than that in normal liver tissues (Supplementary Fig. S1A-B). By analyzing the Cancer Genome Atlas (TCGA) database, the results showed that HCC patients with high ARF6 expression had a poor survival (Supplementary Fig. S1C). Furthermore, ARF6 expression was examined in a tissue microarray from Tongji hospital, which consisted of 169 HCC tissues and adjacent normal liver tissues (Supplementary Table S1). IHC staining and scoring were conducted, and the results showed that ARF6 expression was elevated in HCC tissues, compared with adjacent normal liver tissues (Fig. 1A-B). With clinicopathological features analysis of HCC patients, we found that high level of ARF6 expression obviously correlated with poor tumor differentiation (P = 0.014), incomplete tumor encapsulation (P = 0.005), advanced tumor TNM stage (P = 0.001), and tumor recurrence (P = 0.006) (Supplementary Table S2). Additionally, we found that patients with poor tumor differentiation, advanced TNM stage, tumor recurrence, and incomplete tumor encapsulation had higher ARF6 expression (Supplementary Fig. S1D). By Kaplan–Meier analysis in our patient cohort, we found that patients with high ARF6 expression had low overall survival rate and high recurrence rate (Fig. 1C-D). To further evaluate the clinical significance of ARF6 in HCC, we performed western blot to examine ARF6 expression in a cohort of additional 136 paired HCC tissues and adjacent normal liver tissues, which were obtained from Tongji hospital. We found that ARF6 level was significantly elevated in tumor tissues in comparison with corresponding adjacent non-tumor tissues (Fig. 1e-G and Supplementary Fig. S1E). Together, these findings indicated that ARF6 might play a carcinogenic role in HCC.

### Active ARF6 promotes cell proliferation, colony formation and cell cycle proteins expression in HCC cells

**Fig. 2:**
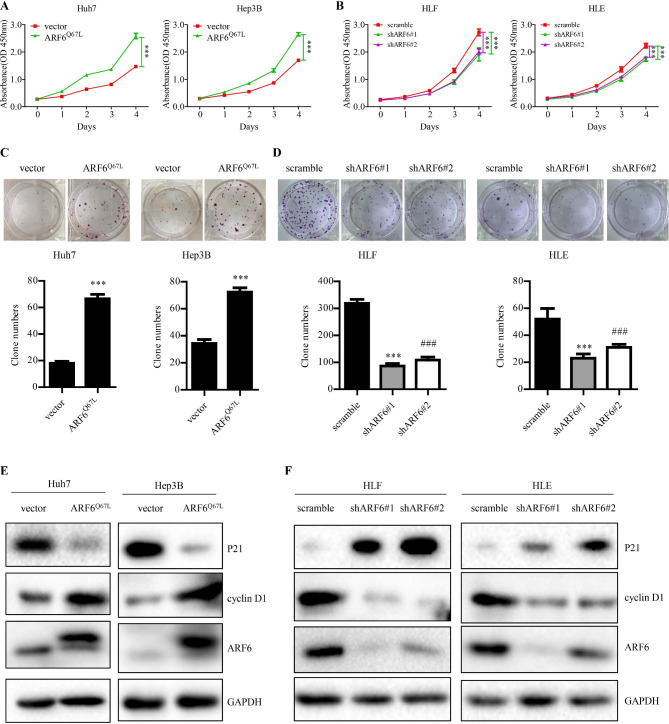
**Active ARF6 promotes cell proliferation, colony formation and upregulated cell cycle proteins in HCC cells. (A, B)** CCK8 assay was performed in indicated cells. **(C, D) C**olony formation assay was performed in indicated cells. Representative images of colonies were shown (upper panel) and the number of colonies were counted (lower panel). **(E, F)** Western blot analysis of the level of cyclin D1, and p21 in indicated cells stably knockdown or overexpression of ARF6, compared with the control groups. ^*^P < 0.05, ^**^P < 0.01, ^***^P < 0.001. ^#^P < 0.05, ^##^P < 0.01, ^###^P < 0.001: the scramble group compared with the shARF6#2 group

Immunofluorescence was performed to show that ARF6 protein localized in the cell membrane and cytoplasm (Supplementary Fig. S2A). Western blot analysis was conducted to test ARF6 expression in some human hepatic and HCC cell lines (Supplementary Fig. S2B). We chose HLF and HLE cells as ARF6 high expression cell line, and Huh7 and Hep3B cells as ARF6 low expression cell line. we stably knocked down ARF6 in HLF and HLE cells by lentiviral transduction of 2 different constructs carrying ARF6 shRNA (shARF6#1 and shARF6#2), and stably overexpressed ARF6^Q67L^ (constitutively active ARF6) [42] in Huh7 and Hep3B cells by retrovirus transduction. We examined ARF6 expression by Q-PCR and western blot analysis (Supplementary Fig. S2C-F). To assess the influence of ARF6 in HCC cell proliferation, we performed CCK8 and colony formation assays. We found that ARF6^Q67L^-overexpressing Huh7 and Hep3B cells had rising cell viability, and elevated number and size of colonies, compared with control groups (Fig. 2A and C). Accordingly, knockdown of ARF6 in HLF and HLE cells obviously inhibited cell viability and the number and size of colonies, compared with their control groups (Fig. 2B and D). Moreover, cell cycle related proteins such as cyclin D1, was upregulated and p21 was decreased in ARF6 overexpressed Huh7 and Hep3B cells (Fig. 2E). In keeping with this, ARF6 knockdown attenuated cyclin D1 expression and increased p21 expression in HLF and HLE cells (Fig. 2F). These results indicated that ARF6 might enhance cell proliferation by influencing cell cycle progression in HCC cells.

### Active ARF6 accelerates tumor growth in HCC in vivo

**Fig. 3:**
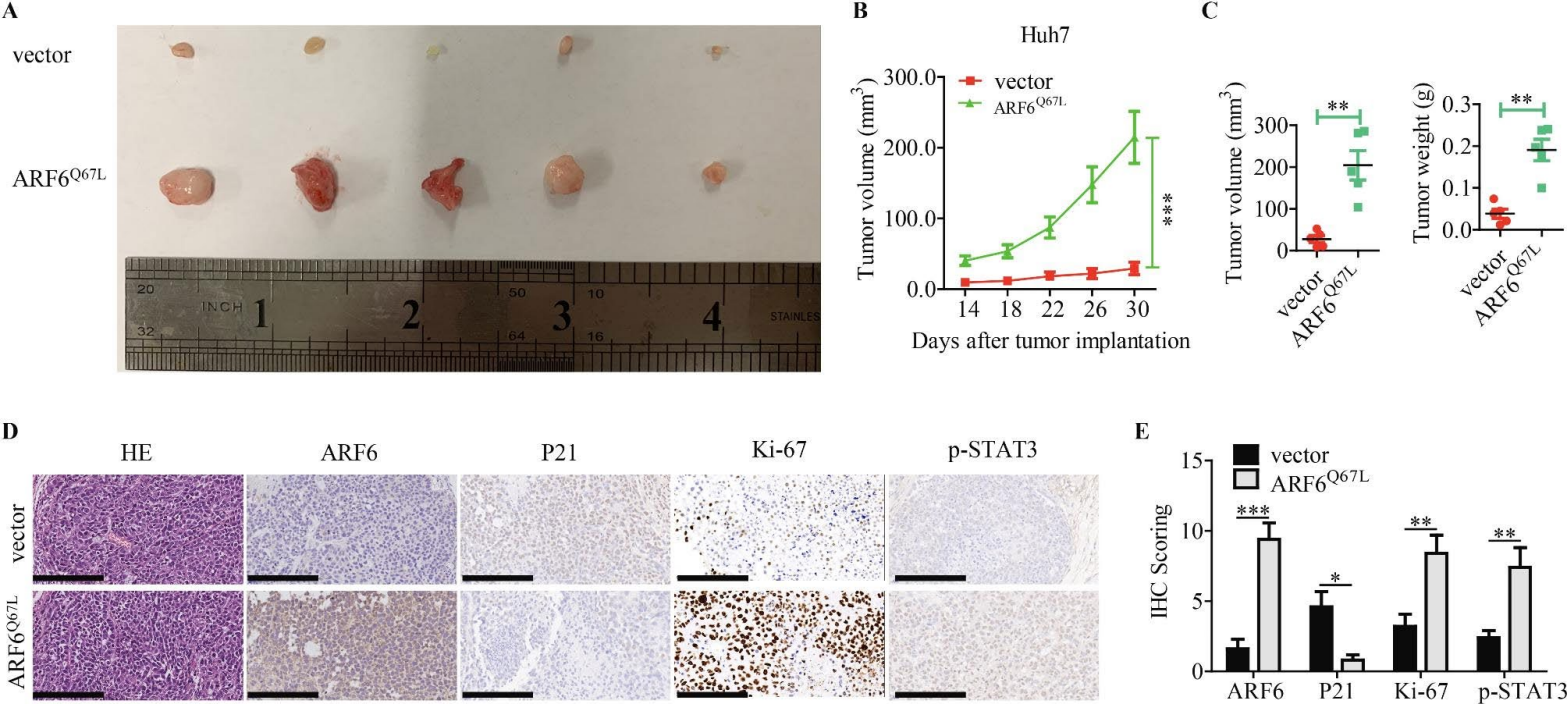
**Active ARF6 accelerates tumor growth in HCC in vivo. (A)** Subcutaneous tumors from Huh7 cells injected into the flank of nude mice. **(B)** Volume of subcutaneous tumors from the two groups were measured at indicated days after injection (n = 5). **(C)** Tumor volume and weight were compared between the two groups after the mice sacrificed. **(D)** The expression of ARF6, P21, Ki67 and p-STAT3 in indicated subcutaneous xenografts confirmed with HE staining was determined by IHC (scale bar: 200 μm). **(E)** expression scoring of above these factors was conducted. ^*^P < 0.05, ^**^P < 0.01, ^***^P < 0.001

To further investigate the oncogenic role of ARF6 in vivo, we applied a xenograft model by injecting HCC cells subcutaneously into nude mice. Huh7 stably overexpressing ARF6^Q67L^ or vector cells were injected into the flanks of nude mice. We measured tumor sizes and weights after inoculation. We found that the tumors generated from Huh7-ARF6^Q67L^ cells were significantly larger and heavier than those generated from control groups (Fig. 3A-C). IHC staining was performed to exhibit the ARF6 overexpressed potency in tumors from Huh7-ARF6^Q67L^ cells. The levels of Ki-67 and p-STAT3 were higher in ARF6^Q67L^ overexpressed tumors, but with lower level of P21, than those in control groups (Fig. 3D-E). These results indicated that active ARF6 enhanced the growth of HCC cells in vivo.

### Active ARF6 promotes STAT3 signaling pathway activation

**Fig. 4:**
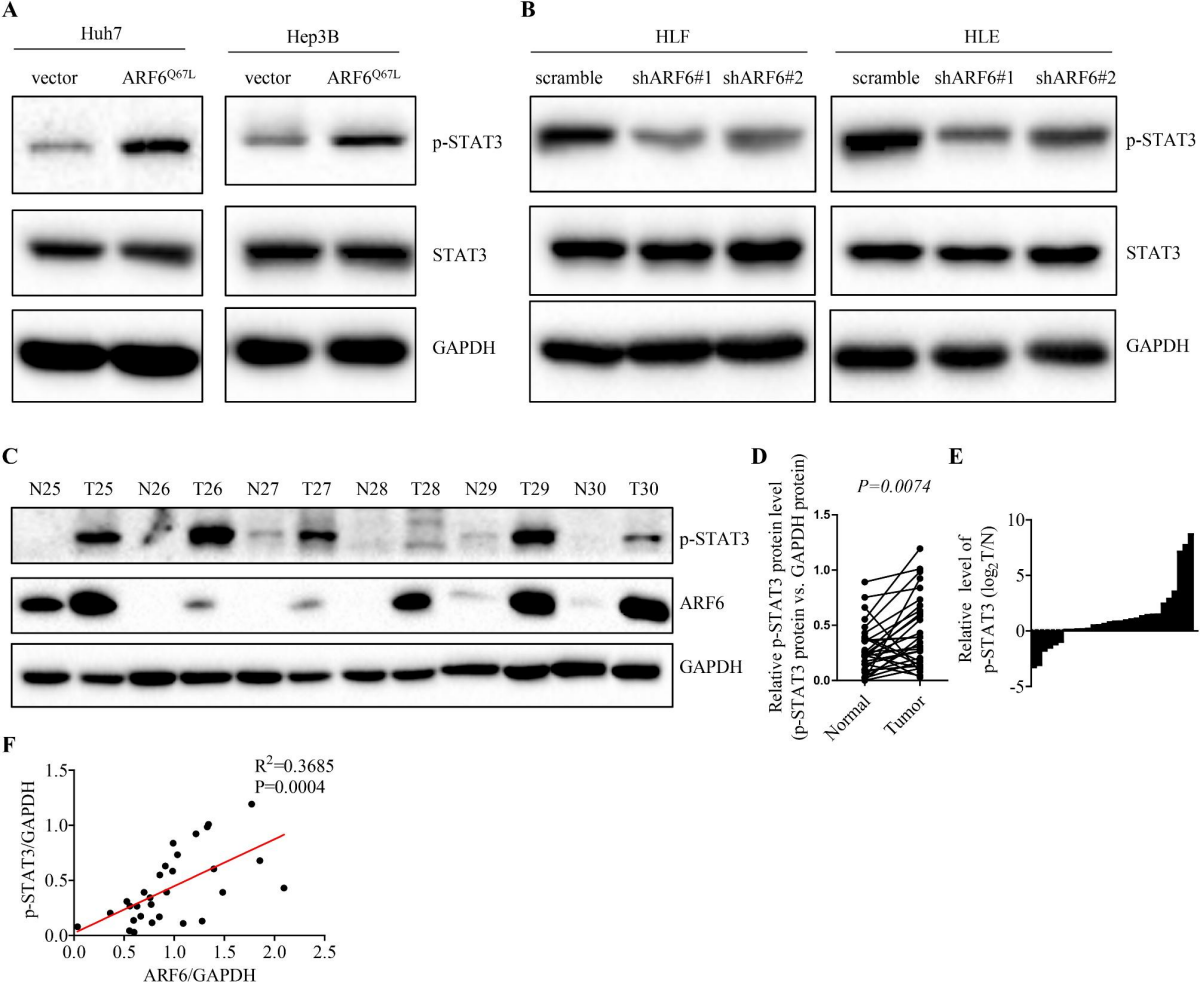
**Active ARF6 promotes STAT3 signaling pathway activation. (A, B)** Western blot analysis of the phosphorylation level of STAT3 in indicated HCC cells. **(C)** The phosphorylation level of STAT3 and ARF6 protein expression were analyzed in the same paired HCC tissues (tumor, T) with corresponding adjacent non-cancerous tissues (normal, N) by western blot. Representative western blot results were shown. **(D)** Statistical analysis showed that phosphorylated STAT3 was upregulated in HCC tissues, compared with that in normal liver, where GAPDH was used as a control. **(E)** Statistical analysis showed that phosphorylated STAT3 bands of HCC tissues were quantified and shown in the bar chart after being normalized to the respective adjacent non-tumor tissues. **(F)** Spearman correlation analysis between ARF6 and phosphorylated STAT3 level (N = 136)

To find the downstream signaling pathways of ARF6 in HCC, we screened some key signaling pathways, such as PI3K/AKT, JAK/STAT3, and p53, and found that the STAT3 pathway was obviously modified by ARF6. In Fig. 4A, active ARF6 overexpression promoted STAT3 phosphorylation in Hep3B and Huh7 cells, without any changes in the level of total STAT3. Furthermore, ARF6 depletion significantly inhibited the phosphorylation of STAT3, compared to the scramble cells (Fig. 4B). To examine the level of ARF6 and STAT3 phosphorylation in paired HCC samples, western blotting was performed. We found that phosphorylated STAT3 and ARF6 were significantly upregulated in tumor tissues compared with corresponding adjacent non-tumor tissues (Fig. 4C-E). Moreover, our results indicated that ARF6 expression was positively correlated with phosphorylated STAT3 level in HCC tissues (Fig. 4F). Together, the data indicated that ARF6 induces STAT3 signaling activation.

### STAT3 signaling is necessary for the oncogenic role of ARF6 in HCC

**Fig. 5:**
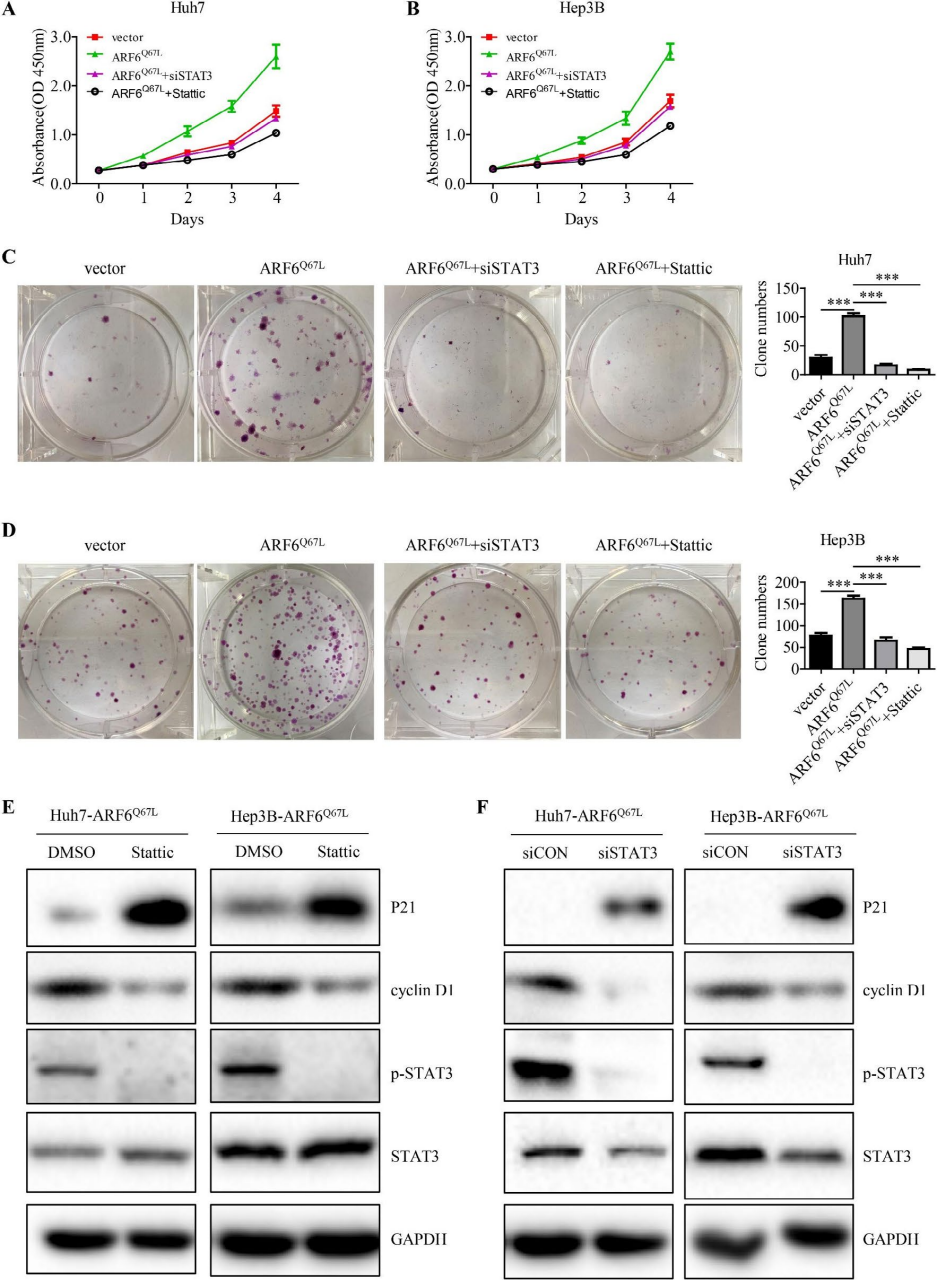
**STAT3 signaling is necessary for the oncogenic role of ARF6 in HCC in vitro. (A, B)** Hep3B and Huh7 stably overexpressing ARF6^Q67L^ cells were transiently transfected siRNA targeting STAT3 or treated with Stattic (2µmol/L). Cell viability of indicated cells were measured by CCK-8 assay. **(C, D)** Indicated cells were subjected to colony formation assay. Representative images of colonies were shown and the number of colonies were counted. **(E, F)** Western blot analysis of phosphorylated STAT3, STAT3, cyclin D1, P21 in indicated ARF6^Q67L^-overexpressed HCC cells transiently transfected siSTAT3 or treated with Stattic. ^*^P < 0.05, ^**^P < 0.01, ^***^P < 0.001

**Fig. 6:**
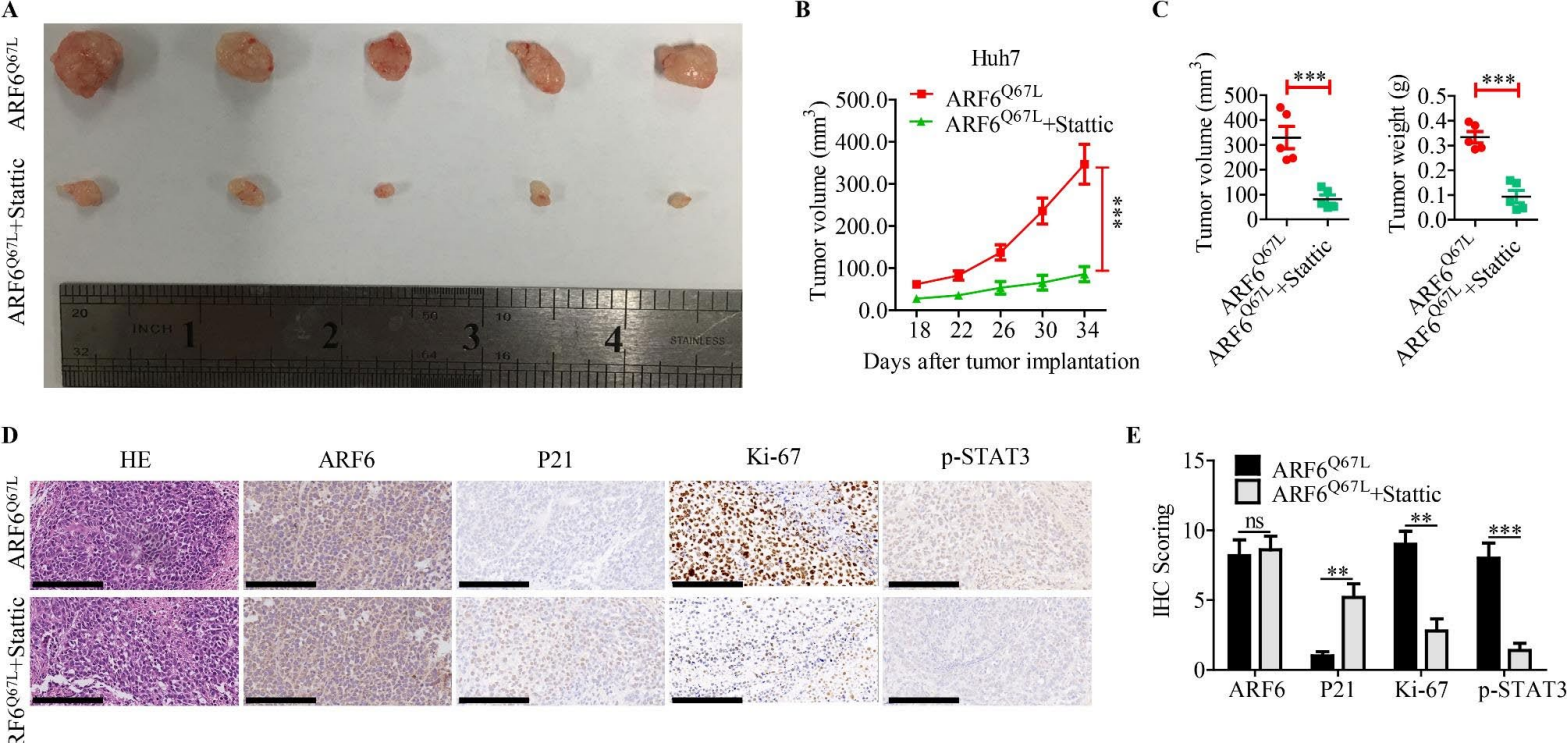
**STAT3 signaling is necessary for the oncogenic role of ARF6 in HCC in vivo. (A)** Huh7 cells stably transfected with control or ARF6^Q67L^ were injected into the fank of nude mice. After the tumors grew to 3–5 mm in diameter, mice were treated with Stattic (50 mg/kg, three times per week for 4 weeks). Representative images of subcutaneous tumors from the two groups were shown. **(B)** Volume of subcutaneous tumors from the two groups were measured at indicated days after injection (n = 5). **(C)** Tumor volume and weight were compared between the two groups after the mice sacrificed. ^*^P < 0.05, ^**^P < 0.01, ^***^P < 0.001. **(D)** The expression of ARF6, P21, Ki67 and p-STAT3 in indicated subcutaneous xenografts confirmed with HE staining was determined by IHC (scale bar: 200 μm). **(E)** expression scoring of above these factors was conducted. ns: no significance, ^*^P < 0.05, ^**^P < 0.01, ^***^P < 0.001

To test whether the effect of ARF6 on HCC cell proliferation was dependent on STAT3 activation, siRNA targeted STAT3 and STAT3 inhibitor Stattic were used. The results showed that siSTAT3 or Stattic treatment obviously inhibited cell proliferation effect induced by active ARF6 overexpression in Hep3B and Huh7 cells (Fig. 5A-D). Western blotting analysis showed that Stattic or siSTAT3 treatment significantly abolished the increased expression of cyclin D1 and rescued the downregulated p21 in active ARF6 overexpressed Hep3B and Huh7 cells (Fig. 5E, F). To evaluate the therapeutic potential and check the role of STAT3 in ARF6-induced tumorigenesis, we conducted subcutaneous xenograft model once again. Nude mice were peritoneally injected with Stattic for one month after tumors growing to 3–5 mm in diameter. Similarly, inhibition of STAT3 by Stattic evidently abrogated the tumor growth induced by active ARF6 overexpression in Huh7 cells (Fig. 6A-C). IHC staining for above xenografts confirmed that Stattic treatment downregulated Ki-67 and p-STAT3 levels in ARF6^Q67L^ overexpressing xenografts, while upregulated the level of P21 (Fig. 6D-E). To sum up, we demonstrated that ARF6 promotes cell proliferation through STAT3 signaling.

## Discussion

As one of the most prevalent and highly malignant tumors, HCC has a high incidence rate and mortality rate [43]. However, the underlying mechanism remains unclear. It is previously indicated that ARF6 is involved in the development of several cancers, such as breast cancer and renal cell carcinomas [44], lung cancer [45], head and neck squamous cell carcinoma [46]. ARF6 is associated with the progress of cancer invasion and metastasis [46–49]. In lung adenocarcinoma, the co-high expression of ARF6, GEP100 and p-EGFR obviously predicts a bad prognosis [45]. Besides influencing tumor metastasis, ARF6 enhances tumor proliferation by activating phospholipase D [50]. In consideration of the above studies, it is proved that ARF6 plays a key role in the development of many tumors. In our research, we found that HCC tissues had higher expression of ARF6 than adjacent normal liver tissues. HCC patients with high ARF6 expression showed aggressive clinicopathological characteristics and poor prognosis. By Cell function and xenograft model assays, we found that ARF6 obviously promoted the proliferation of HCC cells.

It is partly reported about underlying signaling pathways in ARF6-induced cancer progression. EGFR-ARF6 axis regulates the development of tumor invasion and metastasis [46–49]. Some researchers indicate that EGFR-GEP100-ARF6-AMAP1 pathway promotes the development of many tumors [44–46]. It is reported that ARF6/ERK/uPAR signaling pathway boosts the cancer cell invasion and metastasis in breast cancer [51]. In our previous study, DDR1/PSD4/ARF6/ERK signaling axis contributes to the migration, invasion and lung metastasis of HCC cells [42]. It is revealed that EGFR-GEP100-ARF6-Rac-1 axis promotes the invasion and metastasis of breast cancer and HCC cells [52, 53]. WNT5A binds to Frizzled 4-LRP6 complex to activate ARF6, which leads to the separation of β-catenin and N-cadherin, resulting in free β-catenin into nucleus, and ultimately promotes the metastasis of melanoma cells [54]. ARF6 could enhance the internalization of E-cadherin, disrupting cell-cell adhesion, thereby promoting metastasis and proliferation of breast cancer cells [52, 55]. With respect to tumor cell proliferation, a few studies are also reported. ARF6-PLD-mTORC1-S6K1/4E-BP1 axis enhanced tumor cell proliferation [50]. ARF6 accelerates lung tumor cell proliferation through regulating Hh signaling [56]. However, the concrete mechanism of ARF6 in regulating the proliferation of cancer cells, especially in HCC, remains unknown. In our study, the results indicated that active ARF6 boosted HCC cell proliferation by activating the STAT3 pathway.

Signal transducer and activator of transcription (STAT) family is involved in controlling inflammation and immunity reactions dependently of cytokines. It consists of seven members: STAT1, STAT2, STAT3, STAT4, STAT5A, STAT5B and STAT6 [28]. Among them, STAT3 is a transcription factor that has been generally studied in tumor and inflammation. STAT3 activation occurs in most cancers, leading to the expression of various downstream genes responsible for cellular stimulation and influencing cell growth and apoptosis [57]. In HCC, STAT3 has been widely reported to promote the development of HCC [58–61]. Some cytokines and growth factors could activate STAT3 pathway [62], such as IL-6 [63, 64], interleukin-11 [64–66], oncostatin M [67], granulocyte colony-stimulating factor (G-CSF) [68], and epidermal growth factor [63, 69]. However, the concrete mechanism of STAT3 signaling activation in HCC remains vague. In our study, we demonstrated that active ARF6 could accelerate STAT3 signaling activation. Cell function experiments showed that knockdown and inhibition of STAT3 reduced the proliferation of HCC cells induced by active ARF6. Subcutaneous xenograft model had also been performed to confirm that ARF6 boosted HCC cell growth, and the STAT3 inhibitor could reverse the effect. Consequently, STAT3 signaling pathway was necessary for the role of ARF6 in HCC cell proliferation. STAT3 and ARF6 inhibitors might be a potential target for HCC treatment.

## Materials and methods

### Tissue specimens and immunohistochemistry

From January 2006 to December 2012, we collected 169 paired samples of tumor tissues and adjacent normal tissues from HCC patients who experienced tumor resection surgery at Tongji Hospital, Huazhong University of Science and Technology. The pathological examination was used to confirm all HCC tissues. The patients had not experienced systemic or local radiation and chemotherapy before surgery. The patients had undergone no antitumor treatment after surgery. Informed consent forms were signed by HCC patients and the Ethical Committee of Tongji Hospital approved each step. We made HCC staging in accordance with the seventh edition of AJCC (American Joint Committee on Cancer) TNM classification. We made a tissue microarray including 169 HCC cases at Shanghai Biochip Co., Ltd. Shanghai, China. The immunohistochemistry assay was done as previously reported [11]. 3 different pathologists scored the images without knowing about patients’ clinical pathological characteristics. We counted the total score of all images by multiplying staining area percentage score by intensity score, which was previously reported [11]. The cutoff for the definition of low or high expression group was the median value.

### Reagents and antibodies

STAT3 inhibitor Stattic was bought from MedChemExpress, NJ, USA. Polybrene, opti-MEM medium, puromycin, and trypsin-EDTA were acquired as previous mentioned [12]. We mention Lipofectamine 3000 Reagent (Invitrogen, Life Technologies, Carlsbad, CA, USA). We make clear all antibodies used in the study in Supplementary Table S3.

### Cell lines and culture

We obtained cell lines (HL-7702, Alex, HLF, SK-Hep1, HLE, Hep3B, Huh7, MHCC-97 H, MHCC-LM3 and Bel7402) from the Hepatic Surgery Center, Tongji Hospital, Huazhong University of Science and Technology. We bought 293T cells lines from the American Type Culture Collection. Before the study, we examined all cell lines for their authenticity. At 37 °C in 5% CO2 and 95% air condition, we incubated above cell lines in Dulbecco’s Modified Eagle’s Medium with 4.5 g/L glucose (DMEM, Hyclone, Logan, UT, USA), which included 10% fetal bovine serum (FBS, Gibco, North America).

### Plasmids

We obtained pBABE-puro (Plasmid #1764), gag/pol (Plasmid #14,887), pMD2.G(Plasmid #12,259), pLKO.1-TRC cloning vector (Plasmid # 10,878), psPAX2(Plasmid #12,260) from the Hepatic Surgery Center, Tongji Hospital, Huazhong University of Science and Technology. We cloned the human ARF6 cDNA into the BamHI/EcoRI site of the pBABE-puro vector to construct pBABE-Flag-ARF6 plasmid, and identified it by sequencing (TSINGKE, Wuhan, China). We annealed the target double-stranded oligonucleotides (shRNA) sequences and one non-targeting sequence (scramble) and cloned them into the AgeI/EcoRI site of pLKO.1-puro vector, in order to establish pLKO.1-scramble and pLKO.1-shARF6 plasmids. We listed all sequences of target shRNA oligo pairs and siRNA used in the study in Supplementary Table S4. Viral production, infection and establishment of stable cell clones were performed as previously described [70]. We established pBABE-Flag-ARF6^Q67L^ plasmid in accordance with ClonExpress II One Step Cloning Kit and Mut Express II Fast Mutagenesis Kit V2 (Vazyme, Nanjing, China) protocol and confirmed it with sequencing (TSINGKE, Wuhan, China).

### Immunofluorescence

We conducted immunofluorescence assay as previously described [70]. Briefly, cells grew on coverslips in a 24-well culture plate for 12 h, and we fixed cells with 4% paraformaldehyde for 15 min at room temperature. Then cells were permeabilized using 0.5% Triton X-100 for 20 min. After blocking with 5% bovine serum albumin for 1 h, we cultivated cells with indicated primary antibody overnight at 4 °C. Afterwards, we washed the cells three times and cultured them with indicated secondary antibody for 4 h at room temperature. At the end, the slides were incubated with 40, 60-diamidino-2-phenylindole (DAPI, Sigma-Aldrich) for 5 min and visualized under phase-contrast and confocal laser-scanning microscopy.

### Western blot (WB)

We performed western blot assay as shown previously [70]. In brief, cells or tissues were lysed on ice with RIPA lysis buffer, including 1% protease (Roche) and 1% phosphatase inhibitor cocktail (Sigma). We quantified protein samples using BCA assay (Sigma), and separated proteins with sodium dodecyl sulfate-polyacrylamide gel electrophoresis (SDS-PAGE), then transferred proteins onto polyvinylidene fuoride (PVDF) membranes (Millipore). The membranes were blocked with 5% milk, and incubated with indicated primary antibodies, then probed with horseradish peroxidase (HRP)-linked secondary antibodies (Jackson ImmunoResearch, PA, USA). We used ECL for signal detection and western blot images were acquired with Bio-Rad GelDoc system.

### Reverse transcription PCR and real-time quantitative PCR

Cells were lysed with TRIzol Reagent (Invitrogen, Life Technologies, Carlsbad, CA, USA) for total cell RNA extracting. We performed reverse transcription using the QuantScript RT Kit (TIANGEN, Beijing, China) as shown previously [70]. We carried out real-time fluorescence quantitative PCR using the CFX96 Touch™ Real-Time PCR Detection System (Bio-Rad, Hercules, CA, USA) with SuperReal PreMix Plus (SYBR Green) kit (TIANGEN, Beijing, China) as shown previously [70]. All genes expression were normalized to that of glyceraldehyde-3-phosphate dehydrogenase (GAPDH) in the same specimen. All specimens were done independently in triplicate. We used the specific primer pairs to quantify the expression of the genes that encode the proteins. We listed the primers in Supplementary Table S5.

### Cell proliferation assay

We seeded indicated HCC cells in 96-well microplates with the appropriate density per well. After incubated for 0, 1, 2, 3, and 4 d, the cells were treated using Cell Counting Kit-8 (CCK-8, Beyotime Institute of Biotechnology) according to.

manufacturers’ introductions. At the end, the optical density was read at 450 nm using an enzyme-linked immunosorbent assay plate reader (Bio-Tek Elx 800, USA).

### Colony formation assay

We seeded indicated HCC cells on a 6-well plate with the appropriate density per wells. We incubated the cells for about 14 days, and we fixed the colonies using 4% paraformaldehyde and stained them using 1% crystal violet. At the end, we take photos of the plates, and counted the separate adherent colonies larger than 100 μm in diameter.

### In vivo metastasis assay

We conducted animal assays according to Wuhan Medical Experimental Animal Care Guidelines. Male BALB/c (nu/nu) mice (6 weeks old, male, HUAFUKANG BIOSCIENCE CO. INC. Beijing, China) were bred under specific pathogen-free.

(SPF) conditions. We divided the mice into two or more groups at random, and subcutaneously injected indicated HCC cells into the flank of the mice. 14 days later, we began measuring the tumor size every 4 days with digital vernier calipers, and calculated the tumor volume according to the following formula: volume = 1/2× (width^2^ × length). After tumors grew to 3–5 mm in diameter, we peritoneally injected the mice with Stattic (50 mg/kg, three times per week). We sacrificed the mice at appropriate time, and the tumors were visually examined and collected for further analysis.

### Statistical analyses

We conducted data analysis with Prism 5.0 (GraphPad Software, La Jolla, CA, USA) software, and SPSS software (version 21.0, IBM Corp, Armonk, NY, USA). We showed the values as the mean ± SEM from at least done independently in triplicate. We analyzed the difference between two groups by two-tailed Student’s t-test, ANOVA test, a nonparametric test, or a parametric test. We used χ2 test or Fisher’s exact test to analyze categorical data. We analyzed the survival curve between subgroups by Kaplan-Meier and log-rank analysis. We did at least independently in triplicate to guarantee repeatability. We considered a value of P < 0.05 as statistically significance.

### Electronic supplementary material

Below is the link to the electronic supplementary material.

Supplementary Material 1

Supplementary Material 2

Supplementary Material 3

## Data Availability

All supporting data generated or analyzed during this study are available.

## Abbreviations

HCC: Hepatocellular carcinoma
ARF6: ADP ribosylation factor 6
GGT: γ-Glutamyl transpeptidase
TNM: Tumor-Node-Metastasis
Q-PCR: Quantitative real-time PCR
WB: Western blot
IHC: Immunohistochemistry
IF: Immunofluorescence
CCK8: Cell Counting Kit-8
OD: Optical density
OS: Overall survival
DFS: Disease-free survival
