# Supplementary material for: ARF6 promotes hepatocellular carcinoma proliferation through activating STAT3 signaling
Source: Cancer Cell Int. 2023 Sep 16;23:205. doi: 10.1186/s12935-023-03053-y (PMC10505330; doi:10.1186/s12935-023-03053-y)
Supplement: Supplementary file 1 — Supplementary Material 1 [file 12935_2023_3053_MOESM1_ESM.docx]

Supplementary Materials for

**ARF6 promotes hepatocellular carcinoma proliferation through activating STAT3 signaling**

**Supplementary Figure S1**


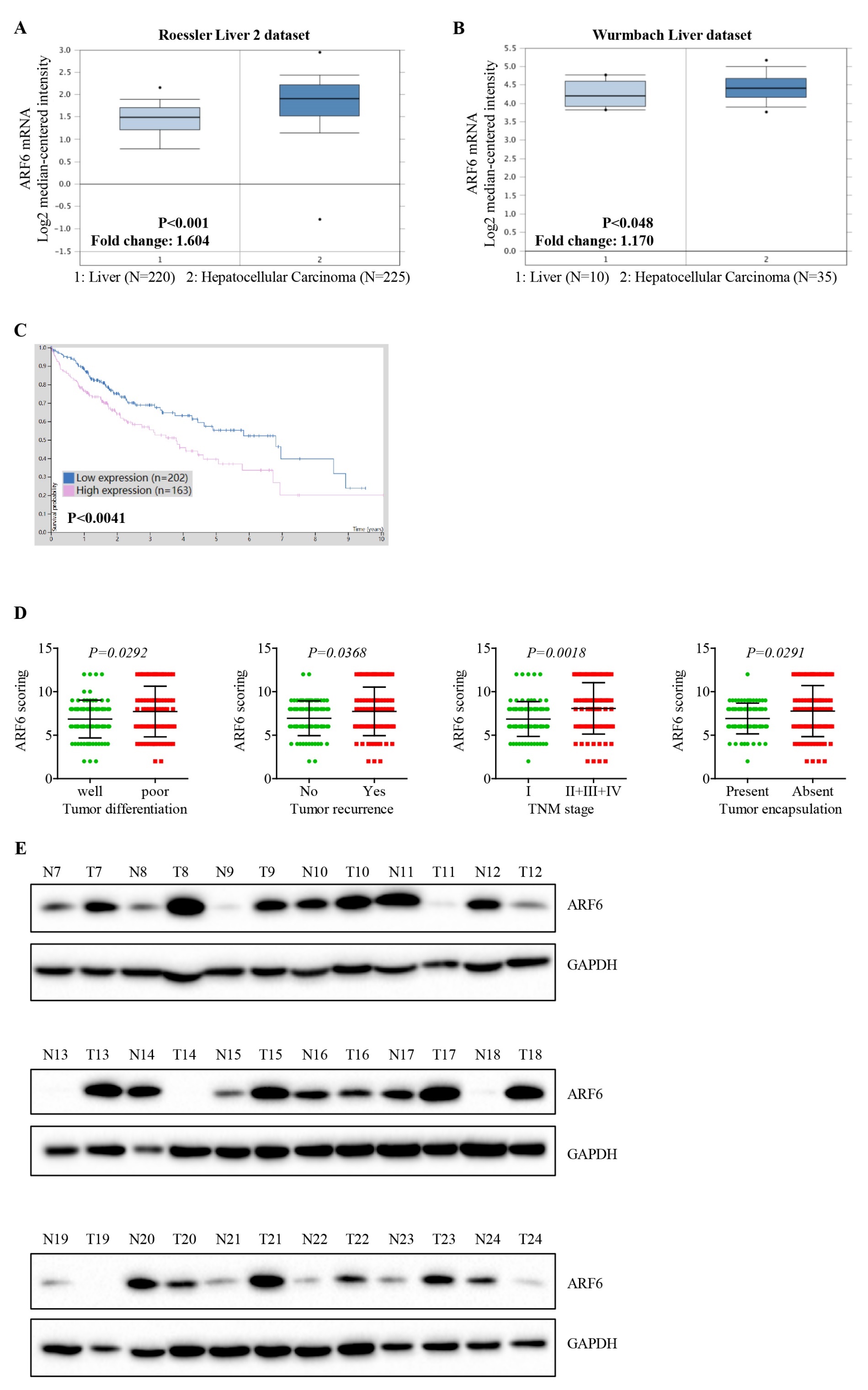


**Supplementary Fig. S1. ARF6 protein is significantly upregulated in HCC tissues and associated with aggressive clinicopathological characteristics. (A, B)** ARF6 mRNA level in normal liver and hepatocellular carcinoma by data analysis in the Dataset from Oncomine database (<https://www.oncomine.org>). **(C)** Kaplan-Meier analyses of the correlations between ARF6 mRNA levels and overall survival of 365 patients with HCC. Data were acquired and evaluated at the website (http:// www.proteinatlas.org); the original data could be found from The Cancer Genome Atlas (TCGA) database. (D) Expression scoring of ARF6 was conducted in different groups of HCC tissues, well tumor differentiation versus poor tumor differentiation (P=0.0292), no tumor recurrence versus tumor recurrence (P=0.0368), TNM stage Ⅰ versus stage Ⅱ-Ⅳ (P=0.0018), and with tumor encapsulation versus without tumor encapsulation (P=0.0291). **(E)** The protein level of ARF6 was analyzed in additional 136 paired HCC tissues (tumor, T) with corresponding adjacent non-cancerous tissues (normal, N) by western blot. Representative western blot results were shown.

**Supplementary Figure S2**


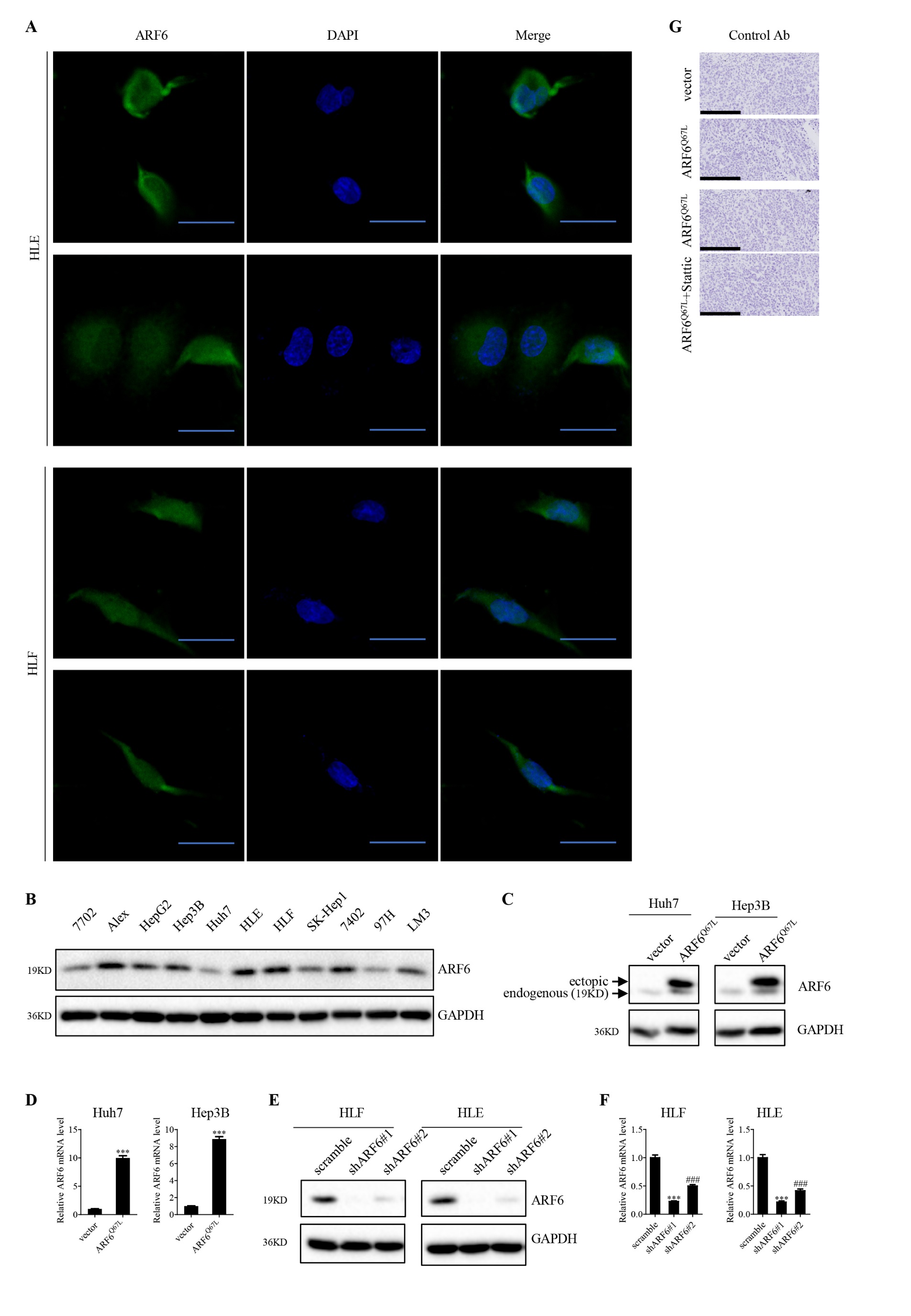


**Supplementary Fig. S2. (A)** Expression of ARF6 (green) in HLF and HLE cells was assessed by immunofluorescence staining. Nuclei were visualized with DAPI (blue) (scale bar: 30μm). **(B)** ARF6 expression level in different liver and HCC cell lines was analyzed by Western blot. **(C, D)** Western blotting and Q-PCR analysis of ARF6 expression in Huh7, Hep3B cell lines stably overexpressed vector and ARF6^Q67L^; **(E, F)** HLF and HLE cells lines stably knocked down of scramble and ARF6. **(G)** Immunostaining of control Ab in indicated subcutaneous tumors (scale bar: 200μm).

| **Supplementary Table S1. Clinicopathologic features of patients with HCC.** | | |
| --- | --- | --- |
| **Clinicopathological Features** | **n=169 Number** | **Percentage (%)** |
|  |  |  |
| ***Sex*** |  |  |
| Male | 145 | 85.80 |
| Female | 24 | 14.20 |
| ***Age(years)*** |  |  |
| ≤50 | 87 | 51.48 |
| ＞50 | 82 | 48.52 |
| ***Serum AFP (ng/ml)*** |  |  |
| ＜400 | 92 | 54.44 |
| ≥400 | 77 | 45.56 |
| ***ALT (ng/ml)*** |  |  |
| ≤75 | 137 | 81.07 |
| ＞75 | 32 | 18.93 |
| ***GGT(u/l)*** |  |  |
| ≤54 | 68 | 40.24 |
| ＞54 | 101 | 59.76 |
| ***HBV*** |  |  |
| Negative | 21 | 12.43 |
| Positive | 148 | 87.57 |
| ***HCV*** |  |  |
| Negative | 167 | 98.82 |
| Positive | 2 | 1.18 |
| ***Differentiation*** |  |  |
| Well/moderate | 88 | 52.07 |
| Poor | 81 | 47.93 |
| ***Tumor size(cm)^#^*** |  |  |
| ＜5 | 75 | 44.38 |
| ≥5 | 94 | 55.62 |
| ***Tumor encapsulation*** |  |  |
| Present | 74 | 43.79 |
| Absent | 95 | 56.21 |
| ***Tumor number*** |  |  |
| Single | 133 | 78.70 |
| Multiple | 36 | 21.30 |
| ***TNM stage*** |  |  |
| I | 91 | 53.85 |
| II+III+IV | 78 | 46.15 |
| ***Recurrence*** |  |  |
| Yes | 89 | 52.66 |
| No | 80 | 47.34 |

^#^ Tumor size was measured by the length of the largest tumor nodule.

**Supplementary Table S2. Correlation between ARF6 Expression with Clinicopathologic Features in HCC (n=169)**

| **Clinicopathological Features** | **Total** | **Relative ARF6 Expression** | | ***P* value** |
| --- | --- | --- | --- | --- |
|  |  | **Low** | **High** |  |
| ***Sex*** |  |  |  |  |
| Male | 145 | 73 | 72 | 0.276 |
| Female | 24 | 9 | 15 |  |
| ***Age(years)*** |  |  |  |  |
| ≤50 | 87 | 40 | 47 | 0.540 |
| ＞50 | 82 | 42 | 40 |  |
| ***ALT (ng/ml)*** |  |  |  |  |
| ≤75 | 137 | 68 | 69 | 0.563 |
| ＞75 | 32 | 14 | 18 |  |
| ***Serum AFP (ng/ml)*** |  |  |  |  |
| ＜400 | 92 | 47 | 45 | 0.537 |
| ≥400 | 77 | 35 | 42 |  |
| ***GGT(u/l)*** |  |  |  |  |
| ≤54 | 68 | 33 | 35 | 1.000 |
| ＞54 | 101 | 49 | 52 |  |
| ***HBV*** |  |  |  |  |
| Negative | 21 | 10 | 11 | 1.000 |
| Positive | 148 | 72 | 76 |  |
| ***HCV*** |  |  |  |  |
| Negative | 167 | 80 | 87 | 0.234* |
| Positive | 2 | 2 | 0 |  |
| ***Differentiation*** |  |  |  |  |
| Well/moderate | 88 | 51 | 37 | **0.014** |
| Poor | 81 | 31 | 50 |  |
| ***Tumor size(cm)^#^*** |  |  |  |  |
| ＜5 | 75 | 38 | 37 | 0.645 |
| ≥5 | 94 | 44 | 50 |  |
| ***Tumor encapsulation*** |  |  |  |  |
| Present | 74 | 45 | 29 | **0.005** |
| Absent | 95 | 37 | 58 |  |
| ***Tumor number*** |  |  |  |  |
| Single | 133 | 65 | 68 | 1.000 |
| Multiple | 36 | 17 | 19 |  |
| ***TNM stage*** |  |  |  |  |
| I | 91 | 55 | 36 | **0.001** |
| II+III+IV | 78 | 27 | 51 |  |
| ***Recurrence*** |  |  |  |  |
| Yes | 89 | 34 | 55 | **0.006** |
| No | 80 | 48 | 32 |  |

NOTE. Statistical significance (P <0.05) is shown in bold

^#^ Tumor size was measured by the length of the largest tumor nodule.

*; Fisher exact test.

| **Supplementary Table S3. Antibodies used in this study.** | | |
| --- | --- | --- |
| Antigens | Manufacturers | Application |
| GAPDH | KC-5G4, Kang Chen Bio-tech, Shanghai, China | 1:10000 for WB |
| anti-rabbit IgG HRP conjugated | Jackson ImmunoResearch Laboratories, Inc. West Grove, PA, USA | 1:5000 for WB |
| anti- mouse IgG HRP conjugated | Jackson ImmunoResearch Laboratories, Inc. West Grove, PA, USA | 1:5000 for WB |
| ARF6 | #5740, Cell Signaling Technology, Beverly, MA, USA | 1:1000 for WB |
| ARF6 | sc-7971, Santa Cruz, CA, USA | 1:50 for IF  1:100 for IHC |
| c-Myc | #5605, Cell Signaling Technology, Beverly, MA, USA | 1:1000 for WB |
| P21 | #2947, Cell Signaling Technology, Beverly, MA, USA | 1:1000 for WB  1:50 for IHC |
| STAT3 | #12640, Cell Signaling Technology, Beverly, MA, USA | 1:1000 for WB |
| p-STAT3 | #9145, Cell Signaling Technology, Beverly, MA, USA | 1:1000 for WB  1:100 for IHC |
| Cyclin D1  Ki-67 | #2978, Cell Signaling Technology, Beverly, MA, USA  #9449, Cell Signaling Technology, Beverly, MA, USA | 1:1000 for WB  1:1000 for IHC |
| IHC, immunohistochemistry; IF, immunofluorescence; WB, Western Blot | | |

**Supplementary Table S4. The sequences of shRNA oligo pairs and siRNA.**

| **Identifier** | **Sequence (5’ to 3’)** |
| --- | --- |
| ARF6-shRNA#1 sense | CCGGCAACAATCCTGTACAAGTTGACTCGAGTCAACTTGTACAGGATTGTTGTTTTTG |
| ARF6-shRNA#1 antisense | AATTCAAAAACAACAATCCTGTACAAGTTGACTCGAGTCAACTTGTACAGGATTGTTG |
|  |  |
| ARF6-shRNA#2 sense | CCGGAGCTGCACCGCATTATCAATGCTCGAGCATTGATAATGCGGTGCAGCTTTTTTTG |
| ARF6-shRNA#2 antisense | AATTCAAAAAAAGCTGCACCGCATTATCAATGCTCGAGCATTGATAATGCGGTGCAGCT |
|  |  |
| STAT3- siRNA | UCUACUUGGCUCCCAACUU dTdT |

**Supplementary Table S5. Sequences of gene-specific primers used for qRT-PCR.**

| Gene | Forward (5’-3’) | Reverse (5’-3’) |
| --- | --- | --- |
| ARF6 | GGGAAGGTGCTATCCAAAATCTT | CACATCCCATACGTTGAACTTGA |
| GAPDH | GGAGCGAGATCCCTCCAAAAT | GGCTGTTGTCATACTTCTCATGG |
