# Supplementary material for: ARF6 promotes hepatocellular carcinoma proliferation through activating STAT3 signaling
Source: Cancer Cell Int. 2023 Sep 16;23:205. doi: 10.1186/s12935-023-03053-y (PMC10505330; doi:10.1186/s12935-023-03053-y)
Supplement: Supplementary file 3 — Supplementary Material 3 [file 12935_2023_3053_MOESM3_ESM.docx]

Supplementary Materials 2 for

**ARF6 promotes hepatocellular carcinoma proliferation through activating STAT3 signaling**

**
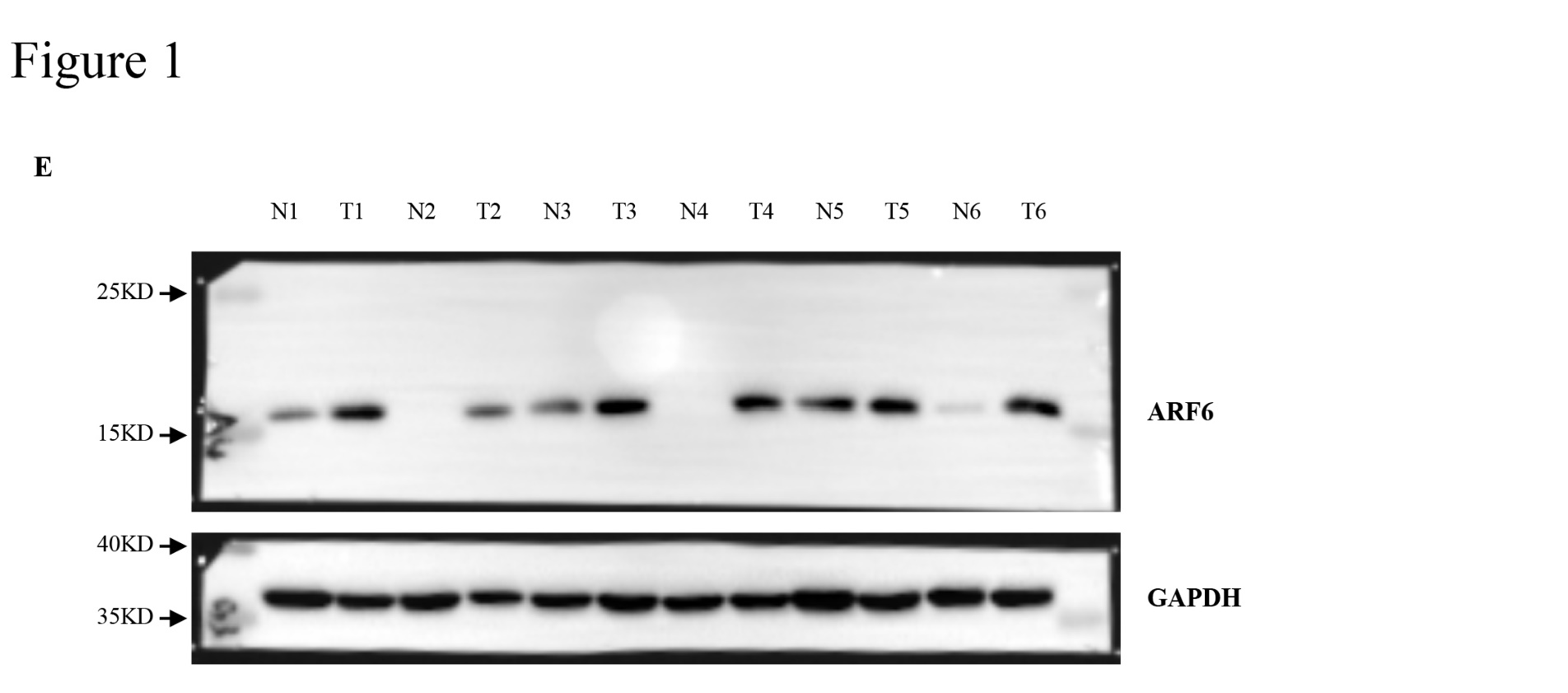
**

**
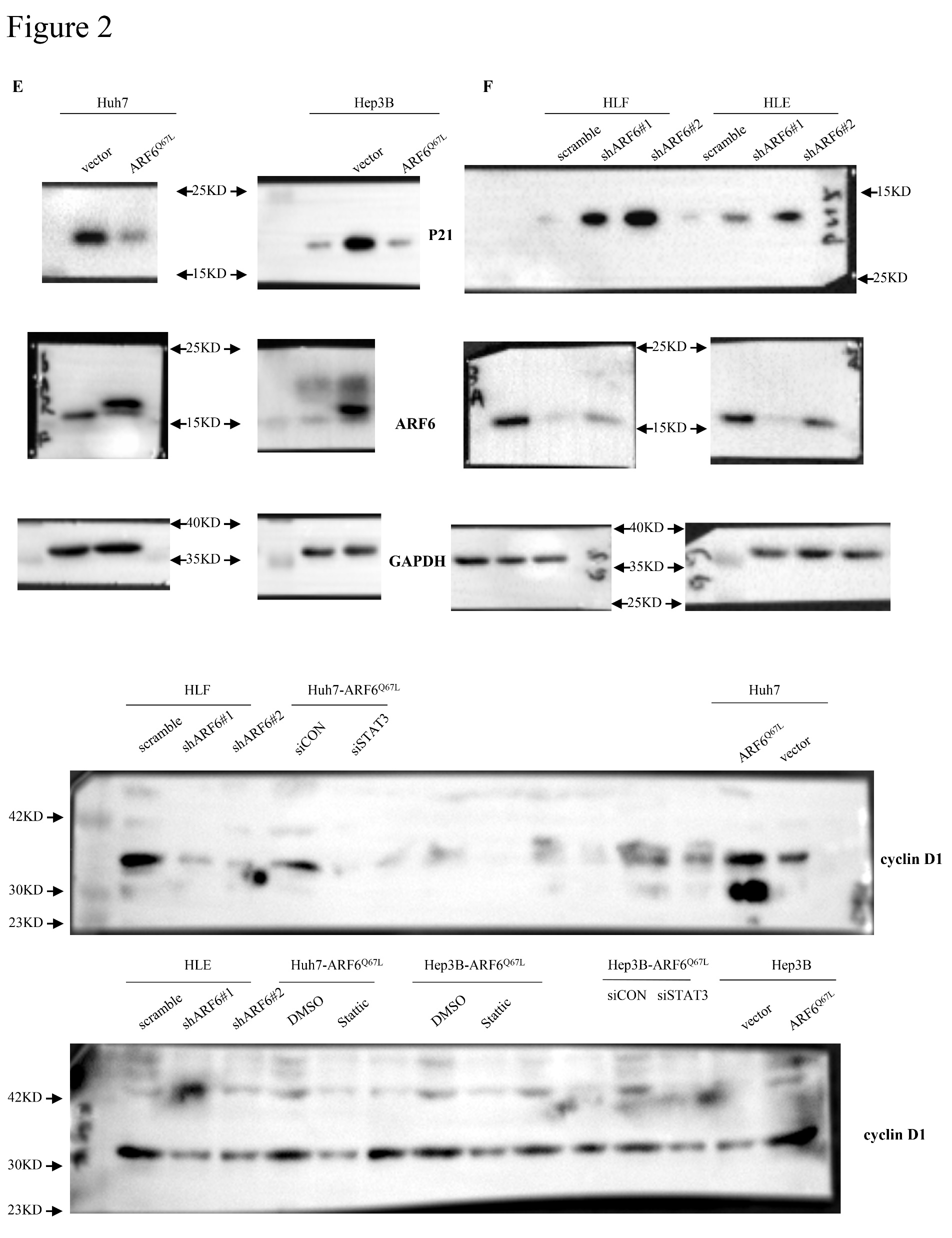
**

**
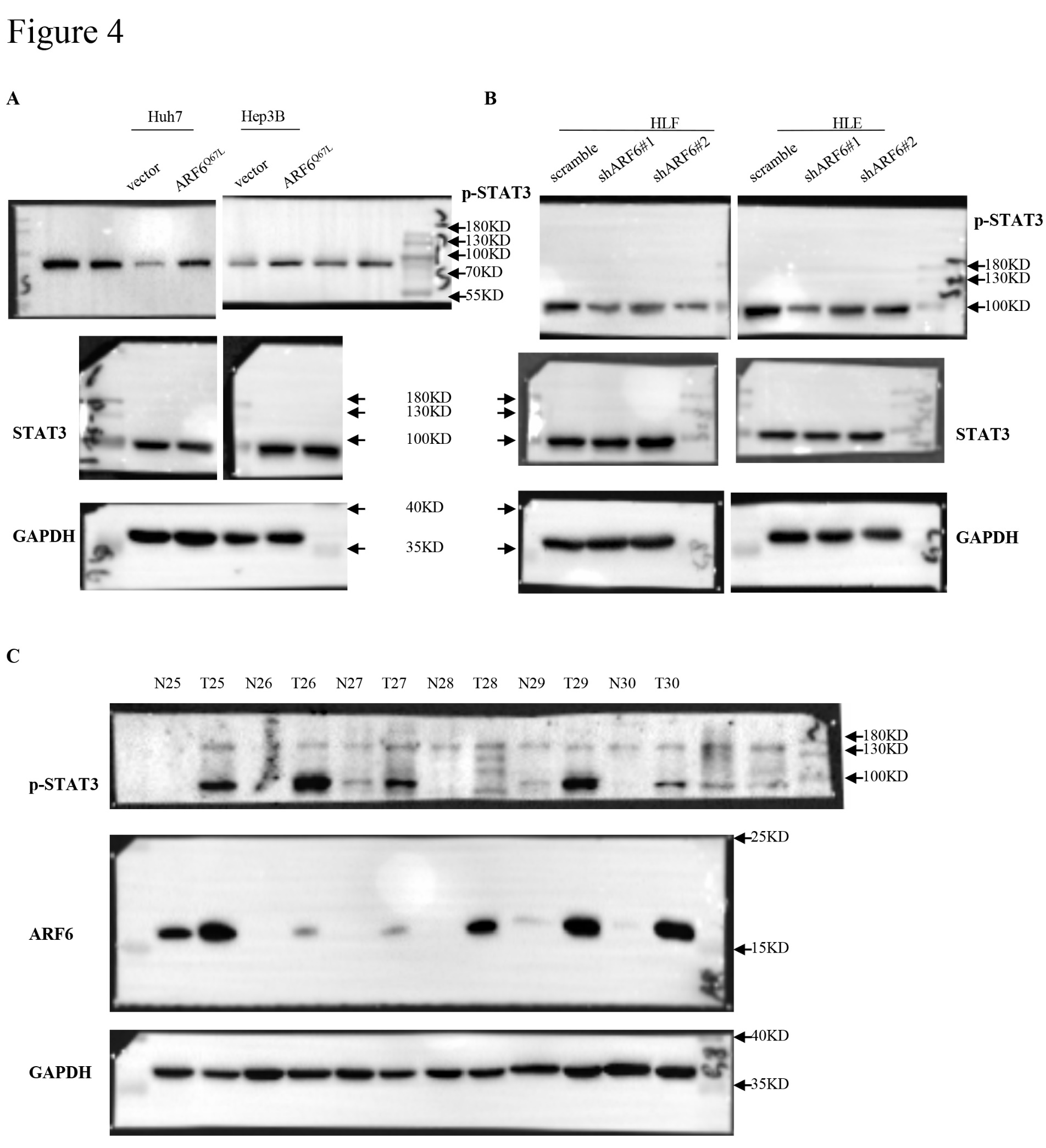
**

**
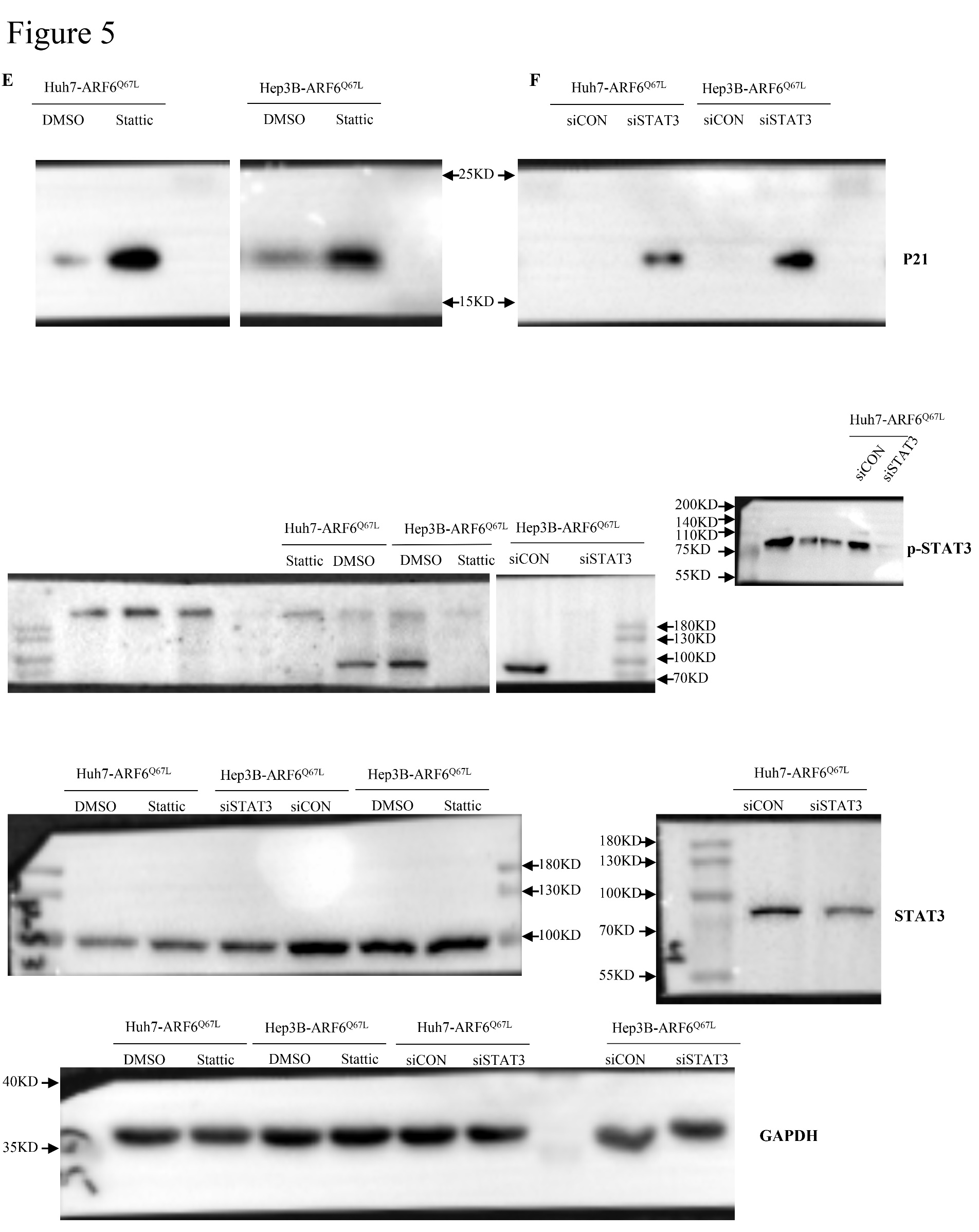
**

**
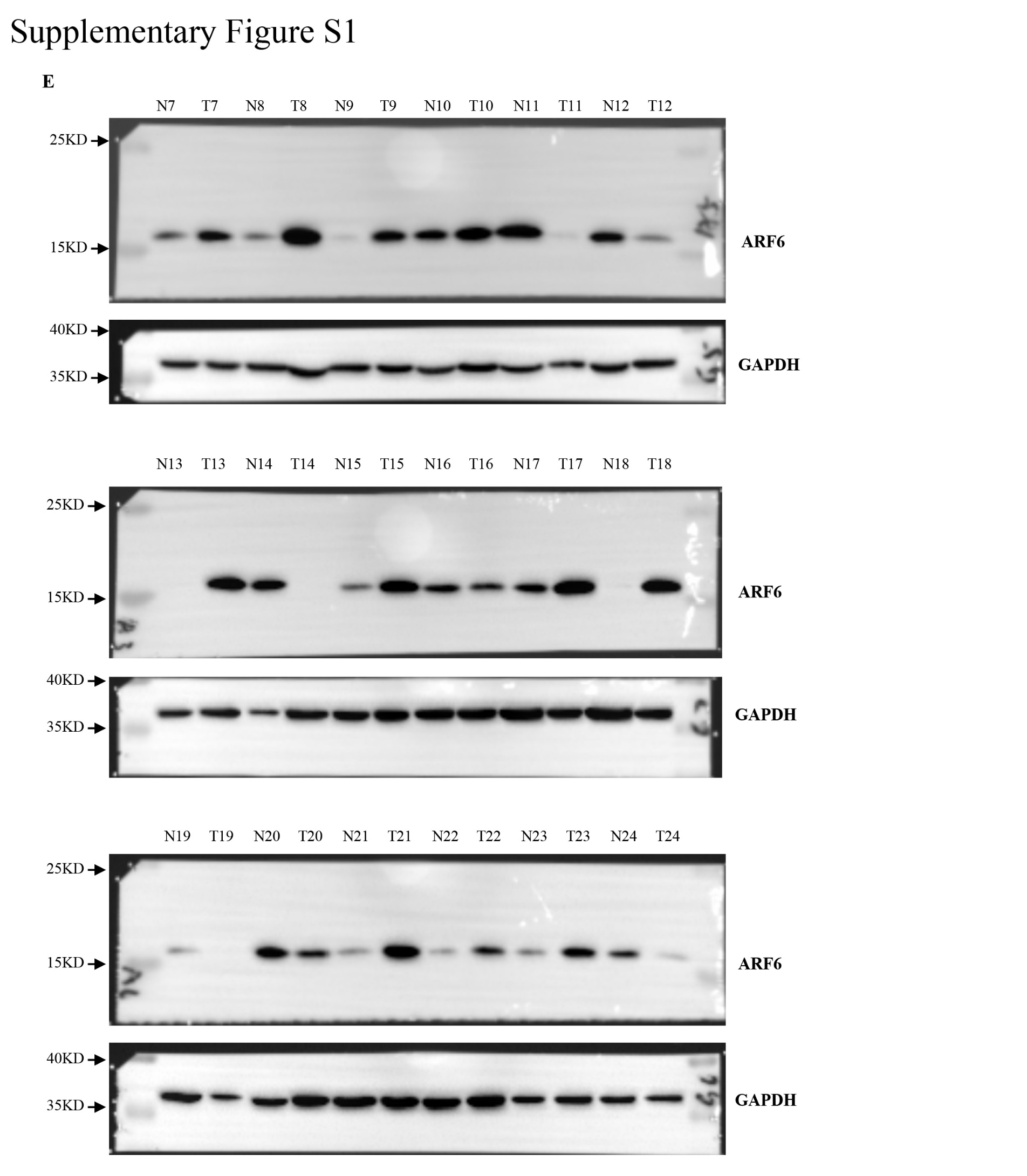
**

**
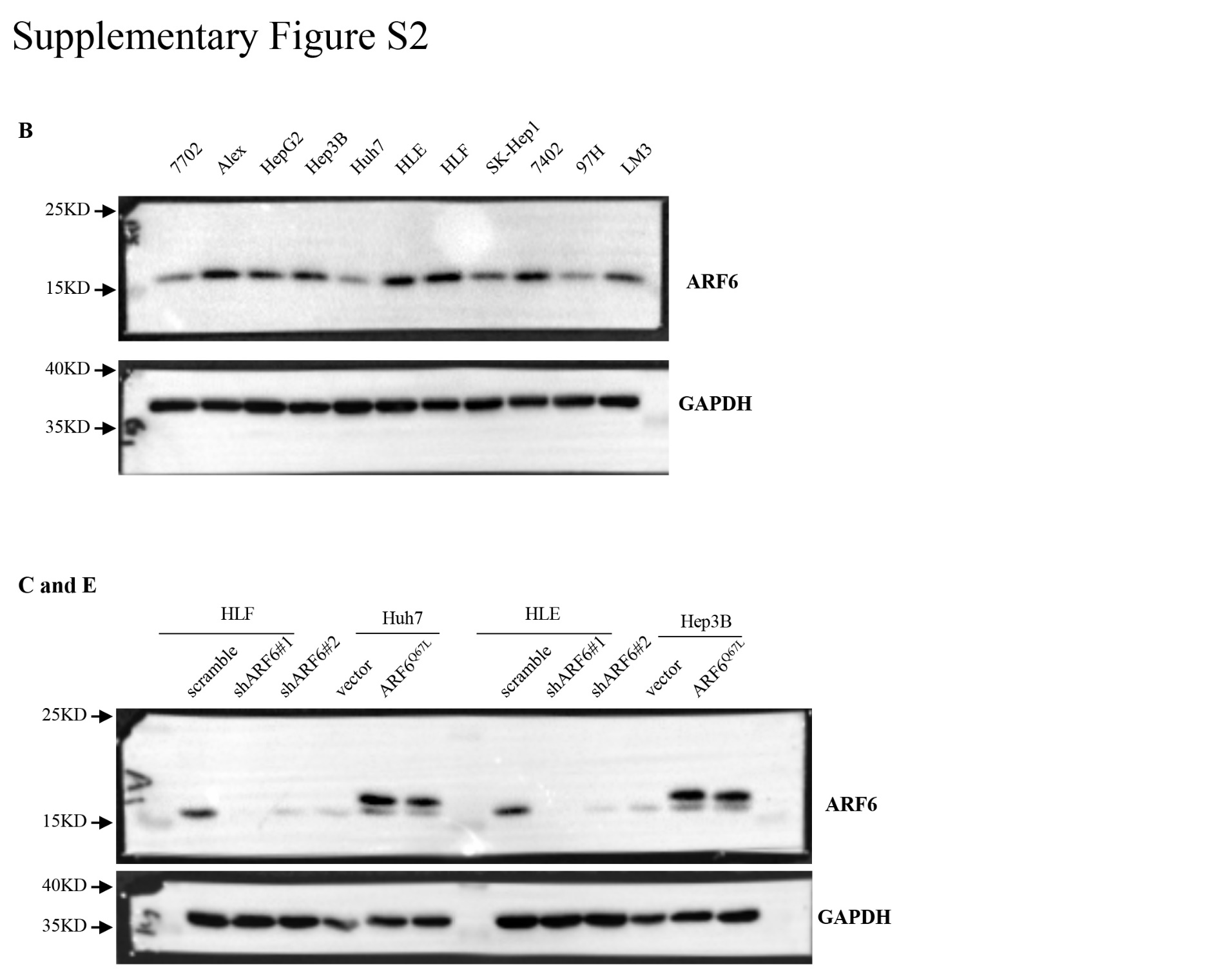
**
